# Supplementary material for: Efficient modification and preparation of circular DNA for expression in cell culture
Source: Commun Biol. 2022 Dec 21;5:1393. doi: 10.1038/s42003-022-04363-z (PMC9772414; doi:10.1038/s42003-022-04363-z)
Supplement: Supplementary file 2 — Supplementary Information [file 42003_2022_4363_MOESM2_ESM.pdf]

Efficient Modification and Preparation of Circular DNA for Expression in Cell Culture (Supplementary Information) - Communications Biology, 2022 -

Roman Teo Oliynyk[1,2*] and George M. Church[1,3]

[1*]Department of Genetics, Harvard Medical School, Boston, MA, USA.
[2]Department of Computer Science, University of Auckland, Auckland, New Zealand.
[3]Wyss Institute for Biologically Inspired Engineering at Harvard University, Boston, MA, USA.

*Corresponding author(s). E-mail(s): roli573@aucklanduni.ac.nz;

Supplementary Note 1. Typical Bacterial Cloning Steps

The preparation of plasmids is labor intensive and time consuming process that typically takes two days [1]. While plasmids can be designed and ordered from contract manufacturers, the cost may be prohibitive, which compels researchers to prepare plasmids themselves. Using as an example a CRISPR/Cas9 or prime editing RNA guide expression vector, the process begins with the initial selection of a suitable plasmid, which may involve ordering it from *Addgene.org* and then modifying it to ligate into the plasmid the guide RNA coding inserts. For each planned genome edit, a researcher must design a specific DNA insert and order it as a DNA fragment from a commercial manufacturer. Then follows the time and labor-intensive process of ligating and cloning this new plasmid. This begins with the ligation of the double-stranded DNA (dsDNA) inserts into a selected plasmid vector using a cloning protocol of choice (e.g., Golden Gate assembly [2] or Gibson assembly [3, 4]). The ligation product is then used in the bacterial transformation protocol using a competent *E. coli* [5]. Then, the transformed *E. coli* is plated onto agar plates overnight. The colonies are

then sequenced and bacteria are grown in liquid culture overnight followed by the recovery of plasmid DNA from bacterial culture [6]. The plasmid concentration is then measured and validated via gel electrophoresis, PCR, or sequencing. At this point, the plasmid expressing the RNA targeting elements is ready for transfection, typically in conjunction with the Cas9-expressing plasmid—unless an all-in-one expression plasmid is used.

Supplementary Note 2. Circular Vector Implementation Specifics

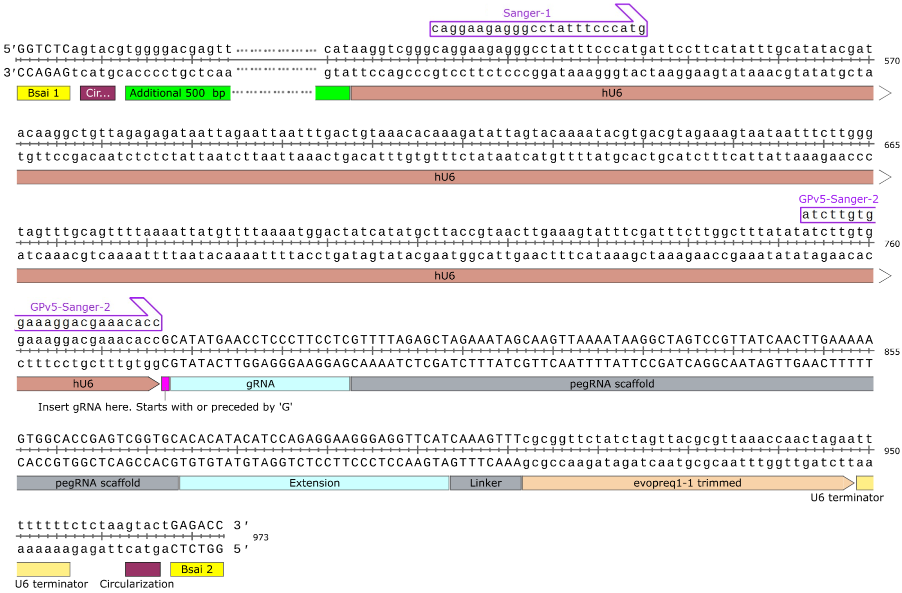

Supplementary Fig. 1 Full example of a *Circular Vector* containing all elements required for PE2max and PE4max. This type of design applies to any short expression vector—and thus not exclusively RNA guides. The end padding is not included because dsDNA was manufactured by Twist Bioscience, with the option to keep the end adapters; otherwise, at least 6 bp of padding would be required on each end. The green interrupted DNA block denotes an optional space for additional padding, which is filled with random well-balanced DNA sequences (exemplified by 500 bp in this case). In this study, this was used to implement the 100, 300, 500, 880, and 1330 bp length extensions.

Supplementary Note 3. Linear Joining of dsDNA Fragments

Joining two or more vectors using Golden Gate protocol reagents can be used as a rapid and fully synthetic preliminary step to join dsDNA fragments with matching overhangs. The majority of commercial suppliers of DNA fragments fail to make dsDNA sequences that contain large repeats. Thus, they would

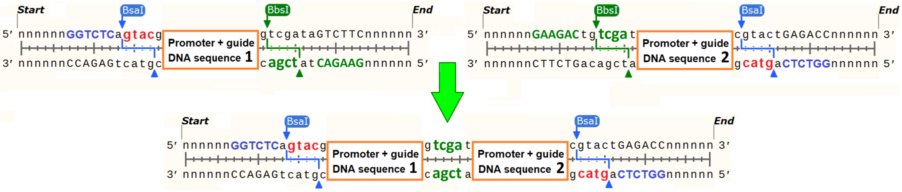

Supplementary Fig. 2 Linear joining of dsDNA fragments as a preliminary step to circularizing dsDNA fragments with long repeats.

not accept an order for such combined fragments. For example, prime editing PE3 and PE5 [7] require two U6 promoters that allow the transcription of two RNA guides. The corresponding constructs are customarily inserted into plasmid vectors using Golden Gate or Gibson assembly, followed by bacterial cloning. In an even more complex scenario, many stages of assembly and bacterial cloning involve approximately 1 week of work and handling [8]. The joining step requires designing and ordering fragments that can be cut with an intermediate type IIS restriction enzyme (i.e., BbsI in the example presented in Supplementary Fig. 2). The joining can be accomplished in a couple of hours, involving 1 hour of a constant 37°C reaction using the reagents listed in Table 4 in the main article while substituting BbsI instead of BsaI. This must be followed by DNA cleanup to discard short DNA end cut-offs by using products such as diluted 1:1 Takara Bio *NucleoSpin® Gel and PCR Cleanup* [9]. Such linear ligation allows us to join linear dsDNA with nearly 100% efficiency, with losses primarily being due to the cleanup using a spin column kit. This preliminary step can then be followed by circularization using the *Circular Vector* protocol, resulting in the entire process being completed many times faster than the many stages of bacterial cloning.

Supplementary Note 4. Reagent Costs Table for a 50 μl Reaction

Supplementary Table 1 Circularization reaction reagent costs.

Component and price	Volume (μl)	Cost
**Step 1: Circularization reaction reagent costs for 6.0 μg of input dsDNA in 50 μl**		
T4 DNA Ligase Buffer (10X B0202S included in M0202L)	5.0	$0 L
T4 DNA Ligase (NEB M0202L 0.250 ml, $202.75)	3.0	$2.43
Bsai-HF2 (NEB R3733L 0.25 ml, $236.00)	3.0	$2.83
ATP (10mM NEB P0756S 1.0 ml, $30.04)	5.0	$0.15
Nuclease-free H_2O (Invitrogen 10977015 500 ml, $27.00)	as needed to 50.0 μl	$0.001
Circularization cost		**$5.41**
**Step 2: T5 exonuclease digestion reaction reagent costs for a 50 μl reaction**		
NEBuffer 4 (10X B7004 included in M0663L)	5.0	$0
T5 Exonuclease (NEB M0663L 0.5 ml, $222.40)	4.0	$1.78
Tris Base 200mM (BB-2686 500ml, $40.43)[1]	2.0	$0.000
Nuclease-free H2O (Invitrogen 10977015 500 ml, $27.00)	as needed to 50.0 μl	$0.002
T5 exonuclease digestion cost		**$1.78**
**Step 3: QIAquick kit DNA purification for a 50 μl reaction**		
QIAquick PCR Purification Kit	1	$2.11
QIAquick kit DNA purification cost		**$2.11**
**Sum total of the *Circular Vector* protocol for a 50 μl reaction (2.0–2.5 μg yield)**		**$9.30**
**Optional: PCR amplification, 75 μl reactions yielding above 6.0 μg of input dsDNA**		
HiFi Hot Start-Readymix (KAPA Biosystems KK2602 6.25 ml, $434.28)	37.5 μl	$2.61
Nuclease-free H2O (Invitrogen 10977015 500 ml, $27.00)	30 μl	$0.002
Two primers combined (Approx 1000 μl, $3.00)	6 μl	$0.015
QIAquick PCR Purification Kit (QIAGEN 28106, 250 reactions $527.50)	1	$2.11
PCR amplification cost		**$4.76**

Supplementary Note 5. Cost Comparison with the Plasmid Preparation by Contract Manufacturers

For comparison, we present the costs of manufacturing classic plasmid based expression vectors for expression of guide RNA and proteins that can be ordered from two commonly used contract manufacturers: Twist Bioscience (Twist) (see at www.twistbioscience.com) and Genscript Biotech Corp (GenScript) (see at www.genscript.com). As usual with contract manufacturers, the pricing is different between these two companies. Both companies allow customers to use stock expression plasmids these companies supply, if they meet the researcher requirements, or submit—in terminology of Twist, onboard—custom plasmids, that these companies will store and clone with inserts designed by researchers.

Twist charges \$500.00 for onboarding of custom vectors, and afterward does not charge extra for cloning a custom plasmid. Twist is charging a minimum fixed price \$80.00 per cloned plasmid with required minimum insert length of 300 bp up to 500 bp. Above 500 bp length, an extra \$0.09 per bp is added. Additionally, Twist charges \$10.00 for an insert with yield in the range of 2–10 μg and \$90.00 for yield of 10–100 μg.

GeneScript accepts custom plasmid submissions for free, and charges \$49 per cloning run afterward. Additionally, GeneScript charges \$159.00 per insert or \$0.35 per bp, whichever is greater. This is the cost for 4 μg with the typical high-copy plasmids, and will cost extra for a larger plasmid delivery.

As noted above, the typical RNA expression plasmids are 2300–3000 bp long, five-fold longer than the *Circular Vector*s we used for the Prime Editing validation in this paper. Thus, if the same molar ratios are desired, the amount of product provided by these manufacturers at 4 μg and a range of 2–10 μg will support fewer edits than our *Circular Vector* yields described in the section above, but will be usually sufficient for a small set of editing experiments.

In summary, for a practically lower but comparable yield, it will cost minimum of \$80 + \$10 = \$90 from Twist (not counting \$500 for a custom plasmid onboarding). And it will cost a minimum \$159 when using a stock expression plasmid from GenScript - and it will cost minimum \$159 + \$49 = \$208 when using a custom plasmid vector. When ordering a large number of plasmids, with negotiation, GenScript may offer slightly lower prices. The *Circular Vector* preparation cost compares favorably to the above prices.

Supplementary Note 6. Gel Electrophoresis Images of the Circular Vector Reaction Product

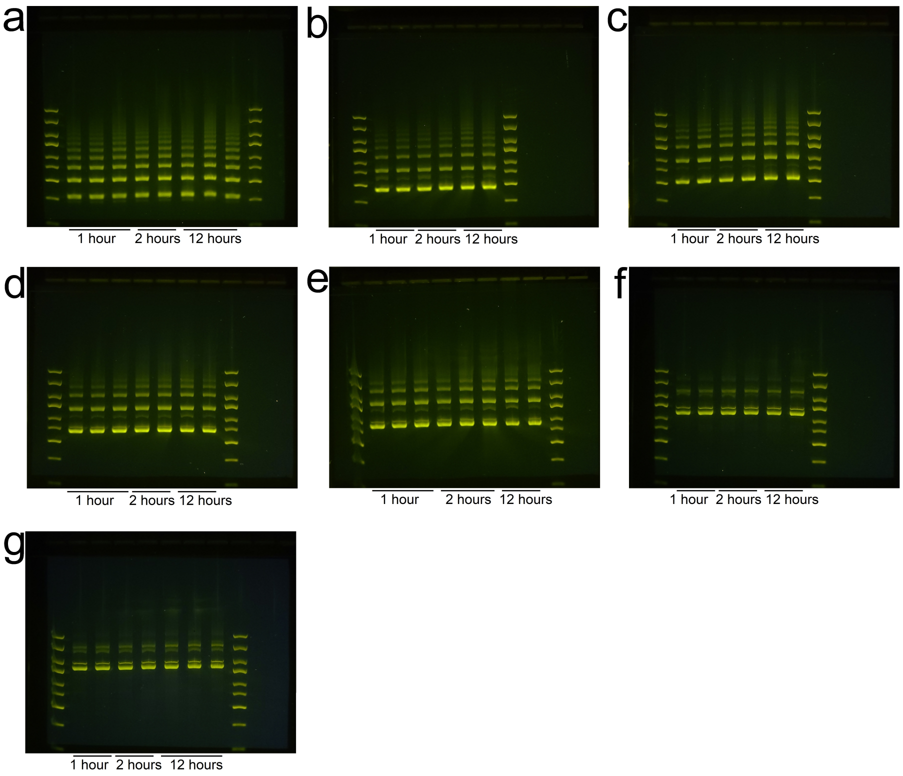

Supplementary Fig. 3 Full set of the unmodified *Circular Vector* gel electrophoresis images used in this study. Images represent the *Circular Vector* lengths as follows: (**a**) 282 bp; (**b**) 452 bp; (**c**) 552 bp; (**d**) 752 bp; (**e**) 952 bp; (**f**) 1332 bp; (**g**) 1782 bp.

Supplementary My References

[1] Casali, N., Preston, A.: E. Coli Plasmid Vectors: Methods and Applications vol. 235. Humana Press, Totowa, N.J. (2003). https://doi.org/10.1385/1592594093

[2] Engler, C., Kandzia, R., Marillonnet, S.: A one pot, one step, precision cloning method with high throughput capability. PloS one **3**(11), 3647–3647 (2008). https://doi.org/10.1371/journal.pone.0003647

[3] Gibson, D.G., Young, L., Chuang, R.-Y., Venter, J.C., Hutchison, C.A., Smith, H.O.: Enzymatic assembly of dna molecules up to several hundred kilobases. Nature Methods **6**(5), 343–345 (2009). https://doi.org/10.1038/nmeth.1318

[4] Addgene: Gibson Assembly Cloning (accessed August 3, 2022). (2022). Addgene. https://www.addgene.org/protocols/gibson-assembly/

[5] Addgene: Bacterial Transformation (accessed August 3, 2022). (2022). Addgene. https://www.addgene.org/protocols/bacterial-transformation/

[6] Addgene: Recovering Plasmid DNA from Bacterial Culture (accessed August 3, 2022). (2022). Addgene. https://www.addgene.org/protocols/purify-plasmid-dna/

[7] Chen, P.J., Hussmann, J.A., Yan, J., Knipping, F., Ravisankar, P., Chen, P.-F., Chen, C., Nelson, J.W., Newby, G.A., Sahin, M., Osborn, M.J., Weissman, J.S., Adamson, B., Liu, D.R.: Enhanced prime editing systems by manipulating cellular determinants of editing outcomes. Cell **184**(22), 5635–565229 (2021). https://doi.org/10.1016/j.cell.2021.09.018

[8] Feng, Y., Liu, S., Mo, Q., Xiao, X., Liu, P., Ma, H.: Enhancing prime editing efficiency and flexibility with tethered and split pegrnas. bioRxiv, 2022–0405487236 (2022). https://doi.org/10.1101/2022.04.05.487236

[9] Takara Bio: NucleoSpin Gel and PCR Clean-Up User Manual (accessed August 3, 2022). (2022). Takara Bio. https://www.takarabio.com/documents/User%20Manual/NucleoSpin%20Gel%20and%20PCR%20Clean/NucleoSpin%20Gel%20and%20PCR%20Clean-up%20User%20Manual_Rev_04.pdf
