# Supplementary material for: Efficient modification and preparation of circular DNA for expression in cell culture
Source: Commun Biol. 2022 Dec 21;5:1393. doi: 10.1038/s42003-022-04363-z (PMC9772414; doi:10.1038/s42003-022-04363-z)
Supplement: Supplementary file 4 — Supplementary Data 1 [file 42003_2022_4363_MOESM4_ESM.zip › Supplementary Data 1 Contents.pdf]

Supplementary Data 1, is composed of the following items and zip fles:

1. *Circularization-Experimental-Data-and-Analysis.xlsx* contains circularization quantifcation data and spreadsheet calculations.
2. *ImageJ-Band-Analysis-Result-SetA.xlsx* and *...-SetB.xlsx* contain two sets of sample ImageJ gel band analysis results.
3. *PrimeEdit-Rate-Spreadsheet.xlsx* contains editing efciency results.
4. *Figures-Source-CSVs.zip* contains input values for fgures in this research.
5. *Gel-Images.zip* contains an extended set of gel photos.
6. *dsDNA-and-Primers.zip* contains the *Circular Vector* designs and primers used for *Circular Vector* validation and prime edit efciency PCR and Sanger sequencing, as well as the sequence fle for the PE4max plasmid with a blasticidin resistance insert (this plasmid is deposited at Addgene as pPE4max-Blasticidin #194905).
7. *PrimeEditSanger-ab1.zip* contains the complete set of Sanger sequencing fles;
